# Etymologia: *Pseudomonas*

**DOI:** 10.3201/eid1808.ET1808

**Published:** 2012-08

**Keywords:** etymologia, Pseudomonas aeruginosa, bacteria, Walter Migula, nosocomial infections, drug resistance, New Delhi metallo-β-lactamase 1, NDM-1

## *Pseudomonas* [soo′′do-mo′nəs]

From the Greek *pseudo* (“false”) + *monas* (“unit”). In 1894, German botanist Walter Migula coined the term *Pseudomonas* for a genus he described as, “Cells with polar organs of motility. Formation of spores occurs in some species, but it is rare.” Migula never clarified the etymology of the term. However, the description of *Pseudomonas* as “false unit” does not make much sense, and an alternative explanation posits that Migula “had not traced directly the Greek ancestry of the name, but had simply created the name *Pseudomonas* for the resemblance of the cells to those of the nanoflagellate *Monas* in both size and active motility.” *Monas* was coined by Danish naturist Otto Friedrich Müller in 1773 to describe a genus of “infusoria” characterized as “*vermis inconspicuous*, *simplicissimus*, *pellucidus*, *punctiformis*” (“inconspicuous worm, simple, transparent, tiny”).

*Pseudomonas aeruginosa* [adj. fem. of *aerūginōsus*] from Latin *aerūgō* (“copper rust or verdigris,” hence green) +‎ -*ōsus* (added to a noun to form an adjective indicating an abundance of that noun) is named for the greenish-blue color of bacterial colonies. The organism has emerged as one of the most serious causes of nosocomial infections.
